# Return to sports after plate fixation of humeral head fractures 65 cases with minimum 24-month follow-up

**DOI:** 10.1186/s12891-017-1532-2

**Published:** 2017-04-26

**Authors:** Philipp Ahrens, Frank Martetschläger, Sebastian Siebenlist, Johann Attenberger, Moritz Crönlein, Peter Biberthaler, Ulrich Stöckle, Gunther H. Sandmann

**Affiliations:** 1 Department of Trauma Surgery, Klinikum rechts der Isar, Technical University Munich, Ismaningerstrasse 22, 81675 Munich, Germany; 2 Sportklinik Stuttgart, Taubenheimstrasse 8, 70372 Stuttgart, Germany; 3 BGU/University of Tuebingen, Schnarrenbergstraße 95, 72076 Tübingen, Germany; 4 German Center for Shoulder Surgery, ATOS Clinic Munich, Effnerstraße 38, D-81925 München, Germany; 5 Department of Orthopaedic Sports Medicine, Klinikum rechts der Isar, Technische Universitaet Muenchen, Ismaningerstr. 22, 81675 München, Germany

**Keywords:** Shoulder, Humerus head fracture, Sports injury, Return to sports after fracture

## Abstract

**Background:**

Humeral head fractures requiring surgical intervention are severe injuries, which might affect the return to sports and daily activities. We hypothesize that athletic patients will be constrained regarding their sporting activities after surgically treated humeral head fractures. Despite a long rehabilitation program physical activities will change and an avoidance of overhead activities will be noticed.

**Methods:**

Case series with 65 Patients, with a minimum follow-up of 24 months participated in this study. All patients were treated using a locking plate fixation. Their sporting activity was investigated at the time of the injury and re-investigated after an average of 3.83 years. The questionnaire setup included the evaluation of shoulder function, sporting activities, intensity, sport level and frequency evaluation. Level of evidence IV.

**Results:**

At the time of injury 61 Patients (94%) were engaged in recreational sporting activities. The number of sporting activities declined from 26 to 23 at the follow-up examination. There was also a decline in sports frequency and duration of sports activities.

**Conclusion:**

The majority of patients remains active in their recreational sporting activity at a comparable duration and frequency both pre- and postoperatively. Nevertheless, shoulder centered sport activities including golf, water skiing and martial arts declined or were given up.

## Background

**Fig. 1:**
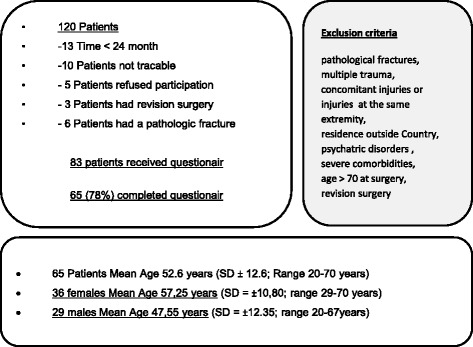
Inclusion/exclusion criteria and biometric data

Fractures of the proximal humerus are common injuries with a vast majority in the older population. 5% of those fractures occur in the proximal third, underlining the enormous importance of a sophisticated approach to this type of injury [3, 24]. Whereas older patients sustain proximal humerus fractures from minor trauma like a falls from a standing height [10], younger patients regularly sustain these injuries following high impact trauma. Due to demographic changes, the incidence is increasing and with the increasing demands of the elderly, a return to daily life activities and especially sporting activities becomes more important [10]. The terminology of fracture classification for this study has been standardized due to the work of Neer (Fig. 1) [23]. Nevertheless, there is a wide range of observer inconsistency concerning the correct fracture type, even when the fracture classification was examined by different experts [22]. For all non-displaced humeral head fractures, the conservative treatment can be seen as gold standard [18, 31]. In contrast, the indications for a surgical interventions are based on the facts such as instability, dislocation or angulations of the fragments compromising the osseous blood supply which in turn postpones the normal healing process up to the development of osteonecrosis [3, 29]. In addition, a displacement of the greater tubercle causing a secondary impingement, is widely accepted as an indication for operative treatment. Many surgical implants have shown their unique potential, such as minimally invasive application procedures. And different nailing or plating systems have unique effects on the on the anatomical reconstruction [12, 15, 29, 30]. However, the number of publications describing various techniques underlines that an anatomical reduction might be more important than a specific implant [26]. A various number of studies focuses on the outcome after surgically treated humeral head fractures, analyzing different approaches (delta-split vs. deltoideo-pectoral) [1, 6] and variable implants (minimally invasive, plates, nails, prostheses) [7, 13, 17, 18]. But nevertheless, not much data concerning the loss of function after humeral head fractures in regard to sporting activities has been generated. Therefore, the aim of this study was to determine the sporting ability of patients who underwent an operative treatment of humeral head fractures using an angular stable implant. In particular, we focused on the participation in different types of sport, their frequency, their duration and intensity. We hypothesized that despite good clinical results, the number and frequency of sports activities would decline and that an avoidance of overhead activities would be noticed.

## Methods

### Patients

**Fig. 2:**
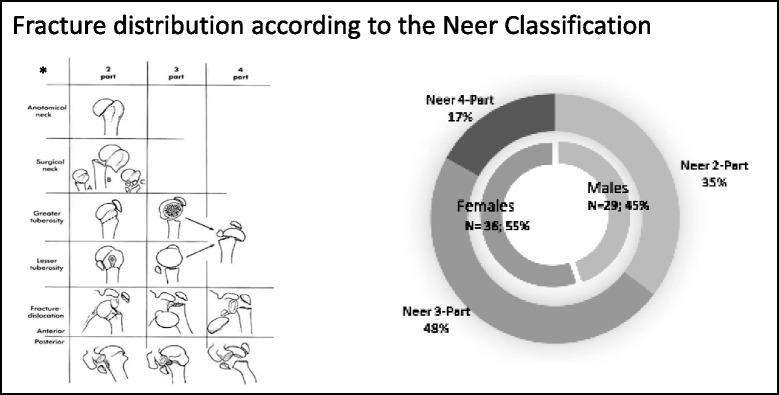
Fracture and gender distribution. Fracture classification according to Neer. The loss of function and disability correlates with the number of fragments and complexity of the fracture. *Neer Charles S. II Displaced Proximal Humeral Fractures Part I. Classification and Evaluation J Bone Joint Surg Am, 1970 Sep 01;52 (6):1077–1089

Between January 2007 and 2010, 120 patients were treated in our hospital because of humeral head fractures. In 13 cases the time between surgery and the evaluation was smaller than 24 months, 10 patients were not traceable for evaluation, 5 patients refused their participation, 6 patients had the fracture due to a neoplastic bone destruction and 3 patients were treated with re- osteosynthesis after therapy failure. At the end 83 patients who underwent surgical treatment for a fracture of the proximal humerus were contacted and 65 patients completed all questionnaires (Fig. 1). Every fracture was characterized by two consultant observers who regularly perform this kind of surgery. The classification was performed according to the Neer Classification (an radiologic approach composed on the basis of four osseous fragments and their regional dislocation (Fragment 1- the lesser tuberosity, 2- the greater tuberosity, 3- the articular surface, and 4- the humeral shaft) [23] (Fig. 2). We excluded patients with conservative treated humeral head fractures (non-displaced fractures) and patients with multiple trauma or concomitant injuries. Furthermore, we excluded patients with attendant injuries in the same extremity, residence outside the country, psychiatric disorders or severe co-morbidities, age over 76 years on the day of surgery, or revision surgery with re-osteosynthesis after initial treatment failure and pathological fractures (Fig. 1). As previously described by Salzmann et al. [25], the survey included a sport and activity questionnaire for the assessment at the time of injury and at the time of the survey in 32 different sports and recreational activities. The questionnaire also inquired the patient’s overall satisfaction with the surgery (very satisfied = 1, satisfied = 2, partially satisfied = 3, not satisfied = 4) and about the use of any pain medication during sporting activity (regularly, occasionally, never). A visual analog scale (VAS) for pain (0 representing “no pain” and 10 representing “maximal imaginable pain”) were used to access the clinical outcome. In addition the functional results were evaluated using the Munich Shoulder Questionnaire (MSQ) as described before by Schmidutz et al. [27]. The Shoulder Pain and Disability Index (SPADI), the Constant-Murley Score (CMS), and the Disabilities of the Arm, Shoulder and Hand Score (DASH) were also ascertained by means of the MSQ. We received approval from our university’s Ethics Committee No. 2993/10.

### Surgical technique

All operations were performed by experienced upper extremity surgeons. The surgical approach used was either the deltoid- pectoral or the delta-split approach depending on the surgeon’s preference. In all patients the minor and the major tubercle were additionally tied up to the plate using Fiberwire cerclages (Fiber wire, Arthrex, Naples, Florida, USA). For post-operative comfort all patients received a sling and the range of motion was limited to 90° of abduction and anteversion for the first 6 weeks. After an X-ray examination 6 weeks post-surgery, unrestricted range of motion was allowed for daily activities. Overhead sporting activities were allowed 3 months after surgery.

### Sports questionnaire

**Fig. 3:**
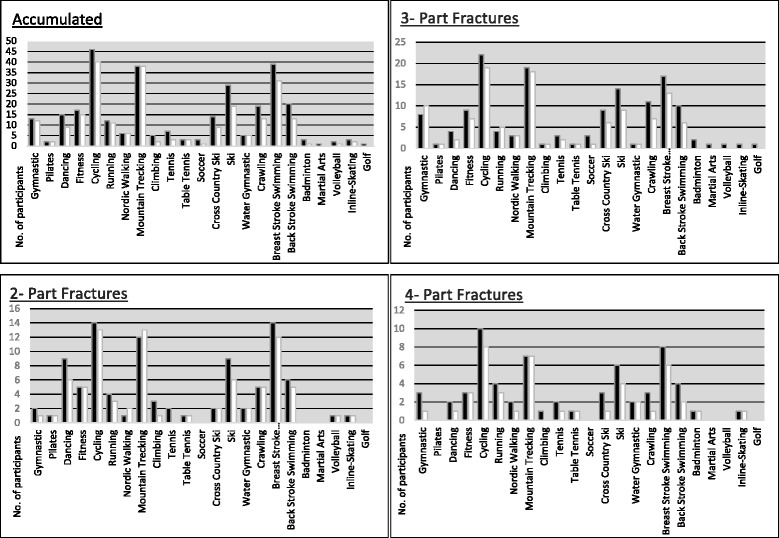
Y- Axis shows the distribution of Sporting participants before and after the surgical intervention, arranged according to the Neer Classification. Dark grey = before surgery, light grey = after surgery. X-Axis shows the different sporting disciplines. There was only increase by 1 participant concerning the Pilates work out in the group of the 3- Part Fractures

All patients were asked about their sporting activity and any existing restrictions. We differentiated whether the restrictions are related to prior trauma or treatment-related. In addition, the query investigated if the patients had to give up, modify or change their particular sporting activities due to the fracture. The second section of the questionnaire included a list of different sporting activities and was used to evaluate the level before and after surgery, the frequency, the intensity and the duration of the sessions per week. The final section of the questionnaire particularly asked for overhead-activity related sport activities. We asked for golf and tennis since these activities put high strains on the upper limb and we wanted a more precise conclusion about this activities (Fig. 3).

### Functional scores

For the evaluation of shoulder function the Munich Shoulder Questionnaire – a validated self-evaluation score [27] -was used. All questionnaires were evaluated directly after their return. All patients were contacted again by phone to clarify any open questions or insufficient markings (Figs. 1 and 2). In detail, the Munich Shoulder Questionnaire (MSQ) as described before by Schmidutz et al.[27] allows the calculation of SPADI [5], the CMS, and the DASH score [2, 4]. The SPADI [4] measures the current shoulder pain and disability in an outpatient setting. The CMS [8] measures the different levels of pain and the ability to carry out normal daily activities. The DASH Score [28] is a self-administered outcome instrument developed to measure self-rated upper-extremity disability and symptoms.

### Statistics

Statistical analysis was performed using the software package SPSS (Version 19, IBM Corp, Somers, New York). All data were tested for normal distribution. Afterwards, normally distributed data were compared using t-tests (MSQ, CS, DASH, SPADI). Non-normally distributed data were compared using Wilcoxon signed-rank and the Mann–Whitney U -tests (paired/unpaired), (Sporting Frequency and Duration). Group data were compared using one-way analysis of variance. Unless otherwise stated, descriptive results were demonstrated as mean standard deviation (STDEV). The level of significance was set at *p* < .05; all confidence intervals are calculated for a 95% confidence level.

## Results

With a recall rate of 78% patients were treated operatively due to fractures of the proximal humerus in our level I trauma center. The average post-operative follow-up was 3.8 years (The mean age at the time of surgery was 52.62 (range: 20–70 years) years with a standard deviation of 12.56 years. The study cohort consisted of 36 females and 29 males; 25 patients had a fracture on the right arm, 40 on the left arm, 59 patients stated their right arm to be their dominant, 6 patients their left arm. According to the Neer classification 23, 2-part fractures (35%; 12f/11 m), 31 3-part fractures (48%; 18f/13 m) and 11 4-part fractures (17%; 6f/5 m) were enrolled (Fig. 2).

### Operative treatment

From the patients included in the study six patients received the Humeral Suture Plate (Arthrex, Corp. Naples, USA), 59 patients received the Synthes Philos Plate (Synthes, Umkirch, Germany). In 15 (23%) cases the deltoid- pectoral approach was used, in 50 (77%) patients the delta split approach was used. At our follow-up, we did not establish any statistically relevant differences between the used approaches.

### Pre-surgical and post-surgical Sporting Frequency and Duration

Throughout the year before the injury all patients were engaged in 26 different sporting disciplines and 23 after surgery. All patients rated themselves as recreational or competitive sportsmen or sportswomen. There was no professional athlete in our study cohort. The participation events or frequency in sports, decreased dependent on the fracture configuration, 2-part (*N* = 21) 3.29 (STDEV 2.26) to 2.57 (STEV 1.86) *p* = 0.07; 3- part (*N* = 29) 2.72 (STDEV 1.71) to 2.66 (STDEV 1.99) *p* = 0.47 and 4-part Fractures (*N* = 11) from 3.36 (STEV 1.69) to 2.82 (STEV 1.94), *p* = 0.18. No significance was found (Sig. *p* = .05). The duration per week (hours/week) changed likewise dependent on the fracture type (*p* = .05). 2-part (*N* = 20) 4.35 (STDEV 2.99) to 3.7 (STDEV 3.01), *p* = 0.17; 3-part (*N* = 28) 4 (STDEV 2.99) to 3.79 (STDEV 3.07), *p* = 0.61; 4- part (*N* = 11) 5.5 (STDEV 3.88) to 5.18 (STDEV 4.09), *p* = 0.17. No significance was found.

### Subjective performance rating

To complete the evaluation, we also questioned the patients about their own subjective performance rating. 22 characterized their individual sporting performance to be decreased as represented by lower MSQ, SPADI, DASH and Constant scores. 37 patients pointed out that there was no change in their sporting ability after the trauma and treatment, one patient refused this part of the questionnaire (Tables 1 and 2).

**Table 1:** Sporting disciplines before and after surgical treatment dependent on the fracture configuration

Sporting Disciplines	Overall		2 Part		3 Part		4 Part	
	before	after	before	after	before	after	before	after
Gymnastic	13	12	2	1	8	10	3	1
Pilates	2	2	1	1	1	1		
Dancing	15	9	9	6	4	2	2	1
Fitness	17	15	5	5	9	7	3	3
Cycling	46	40	14	13	22	19	10	8
Running	12	11	4	3	4	5	4	3
Nordic Walking	6	6	1	2	3	3	2	1
Mountain Trecking	38	38	12	13	19	18	7	7
Climbing	5	2	3	1	1	1	1	0
Tennis	7	3	2	0	3	2	2	1
Table Tennis	3	3	1	1	1	1	1	1
Soccer	3	1			3	1		
Cross Country Ski	14	9	2	2	9	6	3	1
Ski	29	19	9	6	14	9	6	4
Water Gymnastic	5	5	2	2	1	1	2	2
Crawling	19	13	5	5	11	7	3	1
Breast Stroke Swimming	39	31	14	12	17	13	8	6
Back Stroke Swimming	20	13	6	5	10	6	4	2
Badminton	3	1			2	0	1	1
Martial Arts	1	0			1	0		
Volleyball	2	1	1	1	1	0		
Inline-Skating	3	2	1	1	1	0	1	1
Golf	1	0			1	0		

Scoring Evaluation according to the Neer Classification. The loss of function and disability correlates with the number of fragments and complexity of fracture configuration

**Table 2:** Changes in the subjective performance ability, represented in the questionnaires scoring evaluation according to the Neer Classification

Subjective decreased performance *N* = 22							
	MEAN	STDEV	Median	Minimum	Maximum	STDEV of the Mean	Varianz
MSQ	75,91	14,13	80	46	95	3,01	199,61
SPADI (inv.)	78,14	18,74	85	36	100	4	351,36
DASH	19,55	15,03	14,5	0	46	3,2	225,97
Constant	66,32	15,25	69	35	85	3,25	232,61
Subjective unaltered performance *N* = 33							
MSQ	92,85	4,13	94	80	100	0,72	17,07
SPADI (inv.)	97,7	4,53	100	79	100	0,79	20,53
DASH	2,21	3,47	0	0	11	0,6	12,05
Constant	82,55	6,83	83	66	100	1,19	46,63

The loss of function and disability correlates with the number of fragments and complexity of fracture configuration

## Discussion

Fractures of the proximal humerus are demanding and for displaced fractures, the surgical intervention seems to be the appropriate way of treatment [11, 14, 16, 19], although conservative treatment has experienced a renaissance over the last years, especially in non-dislocated fractures [9, 16, 20, 21, 32]. Nevertheless, in fractures with dislocation of the fragments, the locking plate is a widely accepted way of treatment. A variety of studies focusing on the clinical outcome after surgically treated humeral head fractures exists, but there is no reliable data on the return to sports. However, due to demographic changes, the number of proximal humerus fractures is increasing and so are the patient’s demands after surgery. In today’s society, people are increasingly participating in all kinds of sports into a higher age. To our knowledge, this is the first study to examine the return to sports after surgically treated humeral head fractures. Our results support the hypothesis that these kinds of fractures have a temporary impact on the sporting activity and that these injuries lead to an avoiding of overhead sports. Over all 65 patients declared sporting activities before trauma in 26 disciplines. After trauma there were still 23 disciplines left, 88% of the patients indicated to have resumed sports after the end of therapy (Table 1). This shows that with respect to level, frequency, and duration of the sessions, the sports activities after surgically treated humeral head fractures are close to the pre-injury level.

However, the score outcome in the MSQ as well as the calculated results (CMS, DASH, SPADI) were highly dependent on the type of fracture (Table 3) and the shoulder function was reduced tributary to the number of fragments according to the Neer Classification. 2- part fractures had the smallest functional reduction with 87.78 points in the MSQ, the lowest score of the SPADI (8.3), DASH (8.17) and CMS 78.5. The 3- part Fractures showed a MSQ of 87 points and a corresponding slight elevation within the DASH score of 8.5 points. A slight elevation was also found in the SPADI 9.5 and in the CMS 76 points, respectively. The highest functional loss was found in the group of the most complex fracture configuration, the 4-part fractures with an MSQ of 76 points, a DASH of 19.2 and a SPADI of 22 points. The CMS resulted in 65 points. On detailed evaluation we could find that high impact-associated disciplines such as martial arts, waterskiing and golf were abundant. Dependent on the fracture configuration every type lost a fraction in the scores. In the group of the 2- part fractures 21 disciplines were found pre and 19 disciplines post trauma. Tennis and Sailing had been abundant. Looking at the 3 - part fractures 21 of 25 disciplines were left. The group of the 4- part fractures were affected the most from 21 disciplines only 18 remained. Observing the different fracture types in respect to the questioned sporting disciplines the combat associated and shoulder centered disciplines like tennis and golf counted the strongest reduction. Tennis was one of the shoulder centered discipline which showed the greatest amount of reduction, pre Trauma 7 Patients worked out in this field and after surgery 3 (57%) active patients were left. Crawl swimming was trained by 19 Patients before trauma and only 13 patients after surgical treatment (− 32%). Breast stroke swimming was performed by 39 Patients with a reduction of 21% after intervention so 31 patients were left (Fig. 3). The patients in our study reached comparable results to the literature concerning the CMS, the SPADI and the DASH score. In addition, the epidemiologic data were also similar like in recent other studies. Though the mean age with 52.6 years was younger due to the exclusion criteria of the age over 75 years as we wanted to evaluate the shoulder function during sporting activity. Nevertheless, the subjective changes were evaluated and showed that 22 Patients stated a reduction in their sporting ability, while 33 persisted on unaltered abilities (Table 2).

**Table 3:** Scoring Evaluation dependent on the Fracture configuration after surgical intervention following the Neer Classification

MSQ	MEAN	Range	Std Dev	SPADI	MEAN	Range	Std Dev
	85,22	37–100	13,65		88,8	36–100	16,47
2-Part (n23)	87,78	53–100	10,22	2-Part (n23)	91,7	55–100	11,91
3-Part (n31)	86,71	57–97	11,61	3-Part (n31)	90,52	53–100	14,12
4-Part (n11)	75,64	37–96	20,87	4-Part (n11)	77,91	36–100	25,83
Constant				DASH			
	75,43	35–100	13,66		10,22	0–69	14,75
2-Part (n23)	78,48	48–100	10,42	2-Part (n23)	8,17	0–45	10,44
3-Part (n31)	76,06	42–93	12,45	3-Part (n31)	8,55	0–43	13,37
4-Part (n11)	64,64	35–87	17,64	4-Part (n11)	19,18	0–69	22,58

The loss of function and disability correlates with the number of fragments and complexity of fracture configuration. Changes in the Scores can be seen according to the number of fragments following the Neer Classification. (*N* = 65)

One weakness of the study is the absence of a detailed radiographic survey, so that we are not able to report about the development of posttraumatic osteoarthritis and the effects on sports activity. A second limitation of the study is the retrospective design. Patients were asked for sports activities and clinical information that, in some cases, dated back several years’ witch might influence the quality of statements. Also, in total 18 of 80 patients (19%) were lost during the follow-up and 5 patents refused the participation in the study. This might affect the results and produces a possible selection bias. In addition, our study focuses on the outcome after surgically treated patients. It would be desirable to have data on the return to sports after conservatively treated humeral head fractures and a healthy aging population to give athletic patients the best possible advice for their future sports career.

However, we present the results of 65 patients and their return to sports at a minimum follow-up of 24 months postoperatively. Sports activity and shoulder function were assessed by the use of specially designed questionnaires and well established shoulder scores. To our knowledge, this study is the largest series to date with the longest follow-up evaluation of the return to sports after plate fixation of displace humeral head fractures.

## Conclusion

The current study shows that surgically treated proximal humerus fractures seems to be without a significant differences concerning the frequency and intensity of sporting activity. The treatment enables the patients to return to sports, which correlates with the scores used. However, we noticed an avoidance of overhead activities and a change into sporting disciplines upon hip levels. Regarding competition levels, no patient longer participating in sporting competitions after the surgical intervention.

## Abbreviations

CMS: Constant Murley Score
DASH: Disabilities of the Arm, Shoulder and Hand Questionnaire
MSQ: Munich Shoulder Score
SPADI: Shoulder Pain and Disability Index
STEV: Standard deviation
VAS: Visual Analog Scale

## References

[1] Acklin YP, Sommer C (2012). Plate fixation of proximal humerus fractures using the minimally invasive anterolateral delta split approach. Oper Orthop Traumatol.

[2] Angst F, Goldhahn J, Pap G (2007). Cross-cultural adaptation, reliability and validity of the German Shoulder Pain and Disability Index (SPADI). Rheumatology (Oxford).

[3] Bigliani LU, McCluskey GM (1990). 3rd. Prosthetic replacement in acute fractures of the proximal humerus. Semin Arthroplasty.

[4] Breckenridge JD, McAuley JH (2011). Shoulder Pain and Disability Index (SPADI). J Physiother.

[5] Canavese F, Athlani L, Marengo L (2014). Evaluation of upper-extremity function following surgical treatment of displaced proximal humerus fractures in children. J Pediatr Orthop.

[6] Chiewchantanakit S, Tangsripong P (2015). Locking plate fixation of proximal humeral fracture: minimally invasive vs. standard delto-pectoral approach. J Med Assoc Thai.

[7] Clavert P, Adam P, Bevort A, Bonnomet F, Kempf JF (2010). Pitfalls and complications with locking plate for proximal humerus fracture. J Shoulder Elbow Surg.

[8] Constant CR, Gerber C, Emery RJ, Sojbjerg JO, Gohlke F, Boileau PA (2008). Review of the Constant score: modifications and guidelines for its use. J Shoulder Elbow Surg.

[9] Court-Brown CM, Cattermole H, McQueen MM (2002). Impacted valgus fractures (B1.1) of the proximal humerus. The results of non-operative treatment. J Bone Joint Surg (Br).

[10] Court-Brown CM, Garg A, McQueen MM (2001). The epidemiology of proximal humeral fractures. Acta Orthop Scand.

[11] Cuomo F, Flatow EL, Maday MG, Miller SR, McIlveen SJ, Bigliani LU (1992). Open reduction and internal fixation of two- and three-part displaced surgical neck fractures of the proximal humerus. J Shoulder Elbow Surg.

[12] Esser RD (1994). Treatment of three- and four-part fractures of the proximal humerus with a modified cloverleaf plate. J Orthop Trauma.

[13] Foruria AM, Carrascal MT, Revilla C, Munuera L, Sanchez-Sotelo J (2010). Proximal humerus fracture rotational stability after fixation using a locking plate or a fixed-angle locked nail: the role of implant stiffness. Clin Biomech (Bristol, Avon).

[14] Gupta AK, Harris JD, Erickson BJ (2015). Surgical management of complex proximal humerus fractures-a systematic review of 92 studies including 4500 patients. J Orthop Trauma.

[15] Guy P, Slobogean GP, McCormack RG (2010). Treatment preferences for displaced three- and four-part proximal humerus fractures. J Orthop Trauma.

[16] Hauschild O, Konrad G, Audige L (2013). Operative versus non-operative treatment for two-part surgical neck fractures of the proximal humerus. Arch Orthop Trauma Surg.

[17] Jia L, Wang TB, Zhou J, Huang W, Lu H, Jiang BG (2015). Evaluation of the use of structure screw in PHILOS plate for treatment of proximal humerus fracture in Chinese. Beijing Da Xue Xue Bao.

[18] Krettek C, Wiebking U (2011). Proximal humerus fracture: is fixed-angle plate osteosynthesis superior to conservative treatment?. Unfallchirurg.

[19] Kuner EH, Siebler G (1987). Dislocation fractures of the proximal humerus--results following surgical treatment. A follow-up study of 167 cases. Unfallchirurgie.

[20] Launonen AP, Mattila VM (2015). No differences between operative and non-operative treatments of proximal humerus fractures. Evid Based Med.

[21] Maggi G (1984). Elbow hanging cast in the treatment of fractures of the proximal humerus. Review of 614 cases. Chir Organi Mov.

[22] Majed A, Macleod I, Bull AM (2011). Proximal humeral fracture classification systems revisited. J Shoulder Elbow Surg.

[23] Neer CS (1994). 2nd. Fracture classification systems: do they work and are they useful?. J Bone Joint Surg Am.

[24] Palvanen M, Kannus P, Niemi S, Parkkari J (2006). Update in the epidemiology of proximal humeral fractures. Clin Orthop Relat Res.

[25] Salzmann GM, Ahrens P, Naal FD (2009). Sporting activity after high tibial osteotomy for the treatment of medial compartment knee osteoarthritis. Am J Sports Med.

[26] Schmidt-Wiethoff R, Wolf P, Lehmann M, Habermeyer P (2002). Physical activity after shoulder arthroplasty. Sportverletz Sportschaden.

[27] Schmidutz F, Beirer M, Braunstein V, Bogner V, Wiedemann E, Biberthaler P (2012). The Munich Shoulder Questionnaire (MSQ): development and validation of an effective patient-reported tool for outcome measurement and patient safety in shoulder surgery. Patient Saf Surg.

[28] Shimoyama T, Kimura K, Uemura J (2014). The DASH score: a simple score to assess risk for development of malignant middle cerebral artery infarction. J Neurol Sci.

[29] Sturzenegger M, Fornaro E, Jakob RP (1982). Results of surgical treatment of multifragmented fractures of the humeral head. Arch Orthop Trauma Surg.

[30] Sun JC, Li YL, Ning GZ, Wu Q, Feng SQ (2013). Treatment of three- and four-part proximal humeral fractures with locking proximal humerus plate. Eur J Orthop Surg Traumatol.

[31] Young TB, Wallace WA (1985). Conservative treatment of fractures and fracture-dislocations of the upper end of the humerus. J Bone Joint Surg (Br).

[32] Zyto K (1998). Non-operative treatment of comminuted fractures of the proximal humerus in elderly patients. Injury.

